# External iliac artery thrombosis associated with the ilio-inguinal approach in the management of acetabular fractures: a case report

**DOI:** 10.1186/1752-1947-2-4

**Published:** 2008-01-14

**Authors:** Kajetan Klos, Ivan Marintschev, Joachim Böttcher, Gunther O Hofmann, Thomas Mückley

**Affiliations:** 1 Department of Traumatology, Hand and Reconstructive Surgery, Friedrich Schiller University Jena, Erlanger Allee 101, D-07740 Jena, Germany; 2 Institute for Diagnostic and Interventional Radiology, Friedrich Schiller University Jena, Erlanger Allee 101, D-07740 Jena, Germany; 3 Berufsgenossenschaftliche Kliniken Bergmannstrost, Merseburger Straße 165, D-06112 Halle, Germany

## Abstract

**Introduction:**

The ilio-inguinal approach has come to be used routinely in the management of acetabular fractures involving the anterior wall. Thrombotic complications following surgery via this route are a serious, but rare, complication.

**Case presentation:**

We report the case of a 66-year-old male patient who slipped on an icy pavement and fell on his left hip. He sustained a comminuted acetabular fracture (a transtectal T-fracture with an incomplete posterior stem through the ischial tuberosity), and was operated on five days later, via an ilio-inguinal approach. In the recovery room, his left lower limb was found to be cool and pale. Immediate re-exploration showed a left external iliac artery thrombosis, and thrombectomy was performed. In the surgical management of acetabular fractures, thrombosis of a major pelvic artery is a rare but potentially devastating complication. We discuss the possible aetiology (initial vessel trauma versus iatrogenic, intraoperative arterial injury) and pathomechanism, and wish to draw attention to this complication and to recommend ways in which it can be prevented.

**Conclusion:**

We recommend circulation monitoring in patients with acetabular fractures, especially where nerve blocks and/or deep sedation/analgesia have been used. High-risk patients should be identified and subjected to intensive preoperative screening, including ultrasonography and if necessary angiography.

## Introduction

The management of complex pelvic fractures is a major challenge in trauma surgery. In acetabular fractures, surgery via the ilio-inguinal approach is an established and routinely employed technique; alternative approaches are used less frequently. Recognized complications associated with the ilio-inguinal exposure are disruption of the retropubic anastomosis from the femoral to the obturator arterial systems, and damage to the lateral cutaneous nerve of the thigh; [1] major-vessel injuries are rare [2-6]. We describe a case of external iliac artery thrombosis as a rare complication of the ilio-inguinal approach. To our knowledge, this complication has been reported only once before in the current orthopaedic literature [1]. We wish to stress the need, in pelvic surgery, for preoperative circulation screening and close postoperative monitoring of limb perfusion, especially in high-risk patients.

## Case presentation

A 66-year-old male patient slipped on an icy pavement and fell on his left hip, sustaining a comminuted fracture as a result of femoral head impaction into the acetabulum. The fracture was a transtectal T-fracture with an incomplete posterior stem through the ischial tuberosity. The patient had the following comorbidities: atherosclerosis, Type II diabetes, and hypertension.

The patient was referred to the authors' trauma centre. Upon admission to the facility, the patient was put on low-molecular-weight heparin, for thromboembolic prophylaxis. There was no evidence of neurovascular damage at the preoperative physical examination. He was operated on five days after the traumatic event.

**Figure 1:**
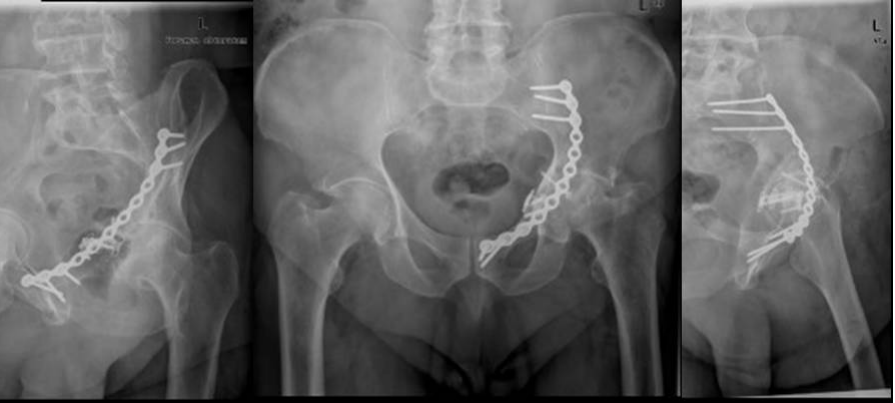
Postoperative radiographs.

The fracture site was approached via the ilio-inguinal route. The external iliac vessel segment was dissected free en bloc, and taken on silicone vessel slings. Anatomical reduction was facilitated by pulling the femoral head laterally, using a Schanz screw as a joystick. Internal fixation was performed with a spring plate for the quadrilateral surface and a curved plate (Matta Pelvic System; Stryker Trauma, Duisburg, Germany) spanning from the internal iliac fossa to the superior pubic ramus (Fig. 1). The procedure did not involve the use of a reduction clamp.

**Figure 2:**
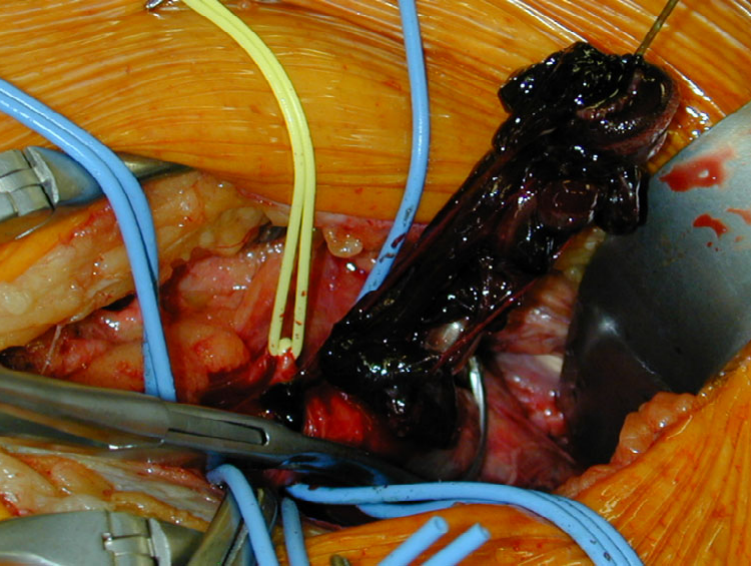
Thrombectomy.

In the recovery room, the patient's left lower limb was found to be cool and pale; no pulses could be palpated. The patient was therefore returned to the operating theatre; the external iliac artery on the operated side was explored and found to be thrombosed (Fig. 2).

Open thrombectomy was performed. The removal of thrombus is shown in Fig. 2, respectively.

**Figure 3:**
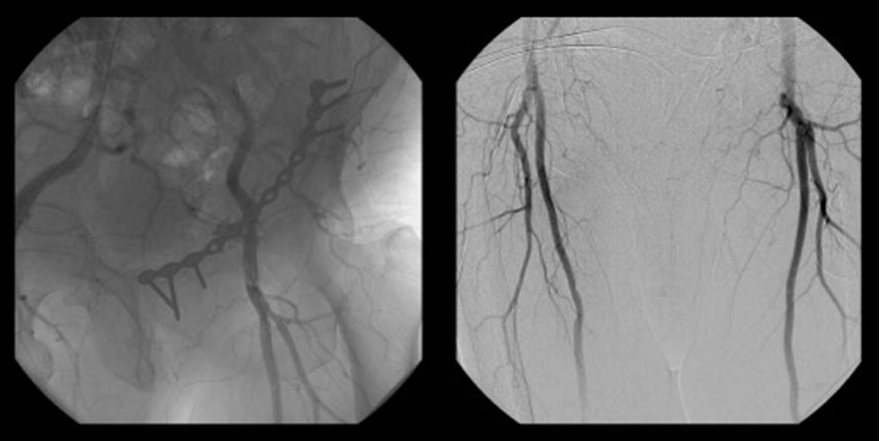
Postoperative angiogram.

Postoperatively, an angiogram was obtained. The perfusion pattern was found to be unremarkable (Fig. 3).

The patient made an uneventful recovery. Postoperatively, an angiogram was obtained as a routin practice. The perfusion pattern was found to be unremarkable At one year, a follow-up investigation with duplex ultrasonography, performed by an experienced radiologist, showed maintenance of the normal pattern.

## Discussion

Perioperative major-artery thrombosis during acetabular surgery is rare. In their description of the ilio-inguinal approach, Letournel and Judet reported one fatal case of arterial thrombosis [4]. To our knowledge, only one other case of ilio-inguinal-approach-associated arterial thrombosis not caused by vascular entrapment between the bone and the implant or in the fracture gap has been published in the recent orthopaedic literature [1]. Thrombotic complications are due mainly to rough handling during fracture reduction, or to malpositioned instruments or implants [1]. With this approach, some vascular structures will, inevitably, be subjected to traction and compression. Probe et al. [1] suggested that these stresses may be responsible for thrombogenesis. The patient described by these authors had further risk factors not encountered in our case: he had sustained high-energy trauma, and had been in traction preoperatively for 26 days; also, a pelvic reduction clamp had been used at surgery. The subject of thromboembolic prophylaxis is not touched upon by Probe et al. [1]

In the present case, the injury resulted from a fall on the hip. The patient was operated on five days after the traumatic event, without any traction having been applied in the interim. In retrospect, this diabetic and hypertensive patient's vascular status must be assumed to have been poor. It is, therefore, conceivable that he suffered a traumatic intimal lesion and/or rupture of an atherosclerotic plaque. It should, however, be borne in mind that thromboembolic prophylaxis (low-molecular-weight heparin) had been administered upon admission, in keeping with the general policy at our centre.

The pathomechanism of traumatic iliofemoral arterial injury has been described by Frank et al. [7] According to these authors, most acetabular fractures result from the femoral head impacting into the acetabulum, or from direct lateral blows to the ilium. At the moment of impaction, the displaced acetabular fragment may exert significant traction force on the distal iliac and proximal common femoral arterial segments. This force will act against the tethering effect of the medially coursing internal iliac and inferior epigastric vessels [7]. The net forces may favour intimal lesions and plaque rupture, and may thus give rise to thrombotic complications. Direct trauma is much less likely, since the vessels are cushioned between the overlying abdominal wall muscles and the underlying iliopsoas groups [7].

Plaque rupture results in exposure of thrombogenic components of the plaque, activation of the clotting cascade, and platelet adhesion; also, procoagulant microparticles are exposed to the blood flow [8-10]. In the case of our patient, the combination of an initial endothelial lesion, intermittent haemostasis during surgery, and further arterial trauma as a result of fracture reduction and vessel retraction, may have been responsible for arterial thrombosis. Obviously, it is impossible to say with certainty which factor was the chief culprit.

Implant malpositioning was ruled out as a causative factor, by postoperative CT scanning.

While the ilio-inguinal approach may, by its very nature, give rise to arterial thrombosis, there do not appear to be any real alternatives in the management of fractures involving the anterior column of the acetabulum. The ilio-inguinal route provides the benefits of a low complication rate, minimal soft-tissue disruption, and good exposure from the anterior column to the sacroiliac joint, to allow anatomical reduction. The rate of heterotopic ossification is extremely low.

The complication described in this report is rare. Good management dictates that the vascular system should be handled as gently as possible. The external iliac vessels should be dissected en bloc, and taken on elastic slings. During surgery, the pulses of the exposed artery should be checked at frequently. Retractor placement should be carefully planned and performed; reduction clamps should not be applied near the vessels; and prolonged traction on the artery should be avoided. If at all possible, preoperative traction should not be applied for long periods of time. Routine pharmacologic thromboembolic prophylaxis is a wise precaution. Careful circulation studies must be performed before and after surgery. Patients with risk factors (such as old age, diabetes, atherosclerosis, or hypertension) should be identified and investigated with ultrasonography. The sophisticated imaging and duplex sonography techniques now available are sufficiently sensitive and specific to allow the individual patient's risk of developing ischaemic events to be assessed. Postoperatively, the patient must be closely observed for vascular impairment, and circulation monitoring must be initiated early after surgery.

## Conclusion

In the surgical management of acetabular fractures, thrombosis of a major pelvic artery is a rare but potentially devastating complication. We recommend circulation monitoring in patients with acetabular fractures, especially where nerve blocks and/or deep sedation/analgesia have been used. High-risk patients should be identified and subjected to intensive preoperative screening, including ultrasonography. The surgical approach depends on the fracture pattern. Intraoperatively, the pulses should be checked frequently, especially during vessel retraction and following the removal of the vascular slings. Postoperatively, the patient should be carefully monitored to detect any signs of iliofemoral arterial impairment. Palpable distal pulses should not, by themselves, be considered as evidence that all is well. If thrombosis is suspected, angiography or (when clinical signs are evident) surgical exploration should be considered. The risk of intimal tears or atherosclerotic plaque rupture as a result of tensile stresses occurring during the traumatic event, during preoperative traction, or during surgical manoeuvres, should not be underestimated.

## Competing interests

The authors declare that they have no competing interests. No financial support from any company was received in the performance of this study, nor do any authors have equity or other financial interest in companies that could benefit commercially from this case report. Written informed consent was obtained from the patient for publication of this case report and any accompanying images. A copy of the written consent is available for review by the Editor-in-Chief of this journal.

## Authors' contributions

KK drafted this paper and assisted in surgeries, IM and TM carried out the operations and diagnosed the described complications, JB carried out the duplex-sonography and participated in the radiologic diagnosis. MT. GH participated in the design of the study and performed the coordination and helped to draft the manuscript. All authors read and approved the final manuscript.

## Consent

A written informed patient consent was obtained for publication of the report and any accompanying images.
